# Escin Ia suppresses the metastasis of triple-negative breast cancer by inhibiting epithelial-mesenchymal transition *via* down-regulating LOXL2 expression

**DOI:** 10.18632/oncotarget.8152

**Published:** 2016-03-17

**Authors:** Yuhui Wang, Xiaotian Xu, Peng Zhao, Bei Tong, Zhifeng Wei, Yue Dai

**Affiliations:** ^1^Jiangsu Key Laboratory of Drug Discovery for Metabolic Diseases, Department of Pharmacology of Chinese Materia Medica, China Pharmaceutical University, Nanjing 210009, China

**Keywords:** escin Ia, triple-negative breast cancer, metastasis, epithelial-mesenchymal transition, lysyl oxidase-like 2

## Abstract

The saponin fraction of *Aesculus chinensis* Bunge fruits (SFAC) could inhibit the invasion and migration of MDA-MB-231 cells. Among which, escin Ia showed more potent inhibition of the invasion than other five main saponin constituents. It selectively reduced the expression of LOXL2 mRNA and promoted the expression of E-cadherin mRNA, and prevented the EMT process of MDA-MB-231 cells and TNF-α/TGF-β-stimulated MCF-7 cells. Moreover, it reduced the LOXL2 level in MDA-MB-231 cells but not in MCF-7 cells. When MCF-7 cells were stimulated with TNF-α/TGF-β, transfected with LOXL2 or treated with hypoxia, escin Ia down-regulated the level of LOXL2 in MCF-7 cells. Meanwhile, escin Ia suppressed the EMT process in LOXL2-transfected or hypoxia-treated MCF-7 cells. Of interest, escin Ia did not alter the level of HIF-1α in hypoxia-induced MCF-7 cells. In TNBC xenograft mice, the metastasis and EMT of MDA-MB-231 cells were suppressed by escin Ia. In conclusion, escin Ia was the main active ingredient of SFAC for the anti-TNBC metastasis activity, and its action mechanisms involved inhibition of EMT process by down-regulating LOXL2 expression.

## INTRODUCTION

Triple-negative breast cancer (TNBC) is an aggressive subtype of breast cancers, which shows higher metastases and relapse rates than other types. Usually, TNBC is treated with chemotherapeutic agents (*e.g.*, doxorubicin, paclitaxel and cisplatin), but its relapse rate is high in the first three years due to the development of drug resistance. In addition, neither tamoxifen nor trastuzumab which can effectively treat other subtypes of breast cancers is effective for TNBC due to the lack of estrogen receptors (ER), progesterone receptors (PR) and human epidermal growth factor receptor 2 (HER2) [1, 2]. Therefore, the conventional chemotherapy and the current targeted therapy are unsatisfactory for the treatment of TNBC, and it is urgent to explore new therapeutic measures with lower toxicity and high efficiency.

Metastasis is a quite complicated process, in which the cancer cells break away from primary tumors, invade surrounding tissues, and migrate into other organs (*e.g*., bone, lung and brain) of the body through blood vessels and lymphatic vessels. Data have indicated that most of patients with TNBC had lymph node metastasis, and died of the complications caused by it. Currently, various promising researches are underway for the identification of new biologics and targeted agents for TNBC treatment, such as those about epidermal growth factor receptor (EGFR) inhibitors, vascular endothelial growth factor (VEGF) inhibitors and lysyl oxidase-like 2 (LOXL2) inhibitors. Prevention of metastasis might be an effective approach for the successful treatment of TNBC [3].

*Aesculus chinensis* Bunge is a deciduous tree that widely distributes in China, and its fruits have long been used for the treatment of various diseases. Evidence indicated that the saponin fraction from *A. chinensis* fruits (SFAC) possesses anti-edematous and anti-inflammatory activities [4, 5]. Also, SFAC exerted anti-cancer activity as shown by inhibition of proliferation and induction of apoptosis in HL-60 cells [6]. Our previous study demonstrated that SFAC could inhibit the invasion (an initiative process of metastasis) of MDA-MB-231 cells (one type of highly aggressive mammary cancer cell) but not their proliferation, apoptosis and angiogenesis (Figures S1 and S2). In the present study, we investigated the main anti-invasion constituent of SFAC, and characterized its action mechanisms for inhibition of TNBC metastasis.

## RESULTS

### Identification of the main anti-invasion constituent of SFAC

**Figure 1:**
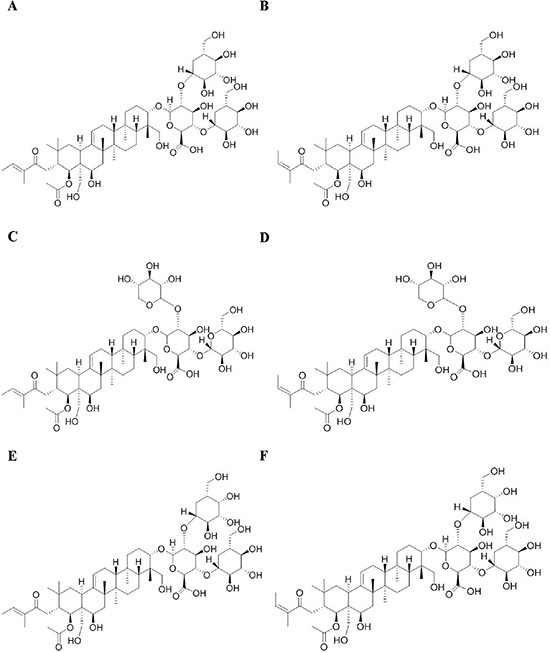
Chemical structures of escin Ia (A), escin Ib (B), escin IIa (C), escin IIb (D), escin IIIa (E) and escin IIIb (F)

**Figure 2:**
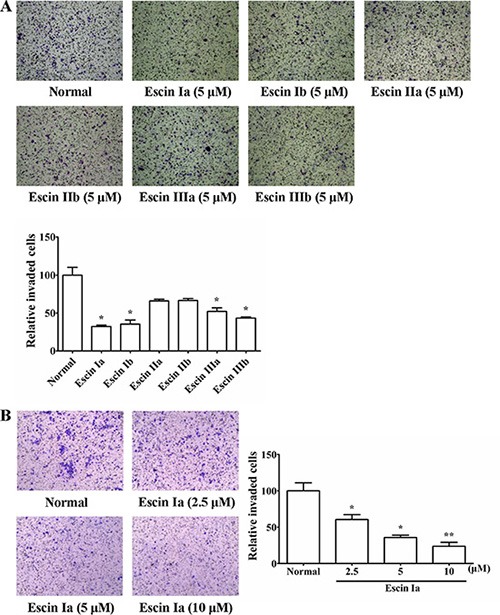
Effect of escin Ia-IIIb on MDA-MB-231 cells invasion (**A**) MDA-MB-231 cells were cultured in chambers with matrigel followed by treatment with escin Ia-IIIb (5 μM) for 24 h. The number of cells invaded through the matrigel of chambers bottom were counted in three different regions. (**B**) MDA-MB-231 cells were cultured in chambers with matrigel followed by treatment with escin Ia (2.5, 5, 10 μM) for 24 h. The number of cells invaded through the matrigel of chambers bottom were counted in three different regions. The data were expressed as the means ± S.E.M. of three independent experiments. *p < 0.05, **p < 0.01 *vs*. normal.

Our previous studies demonstrated that SFAC (5, 10, 20 μg/mL) could inhibit the invasion of MDA-MB-231 cells but not their proliferation, apoptosis and angiogenesis of HUVECs. In order to recognize the major anti-invasion constituent of SFAC, six saponin constituents (escin Ia, escin Ib, escin IIa, escin IIb, escin IIIa and escin IIIb) isolated from it were screened by cell invasion assay (Figure 1A–1F). As shown in Figure 2A, significant invasion of MDA-MB-231 cells was observed in normal group, and escin Ia (5 μM) showed stronger inhibition (inhibition ratio: 68.92%) than escin Ib (inhibition ratio: 63.93%), escin IIa (inhibition ratio: 34.02%), escin IIb (inhibition ratio: 33.14%), escin IIIa (inhibition ratio: 48.39%) and escin IIIb (inhibition ratio: 55.72%). In addition, escin Ia (2.5, 5, 10 μM) inhibited the invasion of MDA-MB-231 cells by 39.46%, 64.22% and 76.23%, respectively (Figure 2B). The findings in association with the fact that the content of escin Ia in *A. chinensis* is much higher than escin Ib suggested that escin Ia was the major anti-invasion active constituent of SFAC [7].

### Escin Ia inhibited invasion process of MDA-MB-231 cells by down-regulating the expression of LOXL2 and up-regulating the expression of E-cadherin

**Figure 3:**
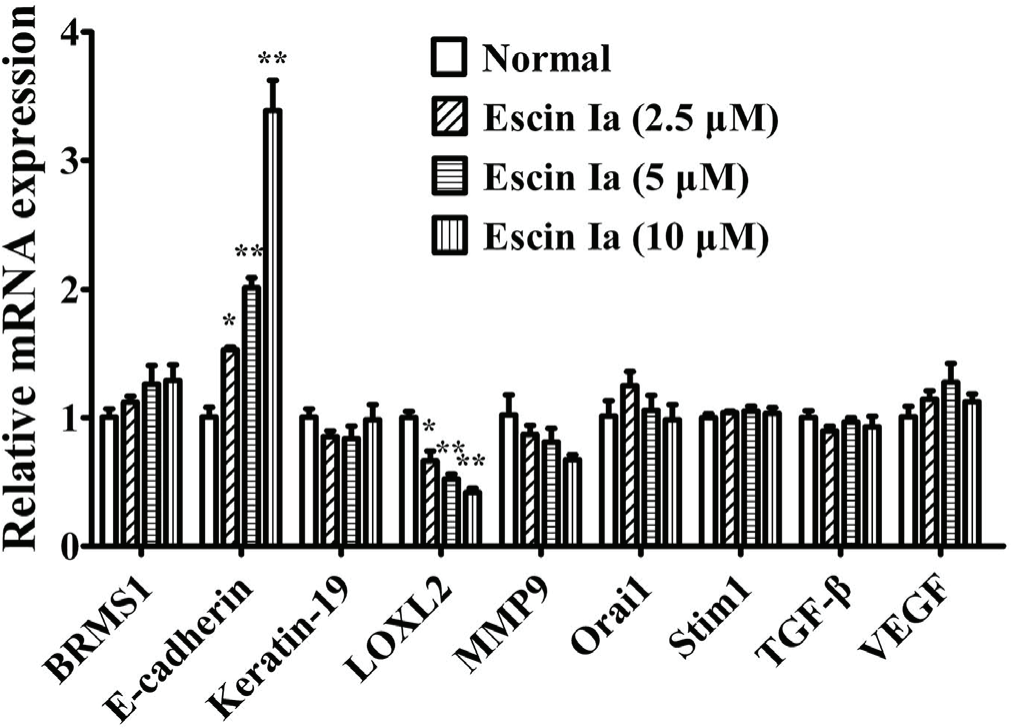
Effect of escin Ia on invasion-related factors in MDA-MB-231 cells MDA-MB-231 cells were treated with escin Ia (2.5, 5, 10 μM) for 24 h, and the mRNA expressions of breast cancer metastasis suppressor 1 (BRMS1), E-cadherin, Keratin19, lysyl oxidase-like 2 (LOXL2), metal matrix proteinase 9 (MMP9), calcium release-activated calcium channel protein 1 (Orai1), stromal interaction molecule 1 (Stim1), transforming growth factor (TGF-β) and vascular endothelial growth factor (VEGF) were measured by using Q-PCR assay. Gene expressions were normalized to GAPDH. The data were expressed as the means ± S.E.M. of three independent experiments. *p < 0.05, **p < 0.01 *vs*. normal.

The invasion of tumor cells could be regulated by many factors such as BRMS1, E-cadherin, Keratin19, LOXL2, MMP9, Orai1, Stim1, TGF-β and VEGF [8–15]. To identify the mechanisms underlying anti-invasion effect of escin Ia, its effect on the expressions of the factors mentioned above were studied. As shown in Figure 3, escin Ia (2.5, 5, 10 μM) obviously down-regulated the LOXL2 mRNA expression and up-regulated the E-cadherin mRNA expression of MDA-MB-231 cells. In addition, MMP9 mRNA expression in MDA-MB-231 cells was moderately down-regulated by escin Ia (10 μM) treatment. In contrast, escin Ia only showed slight effect on the expressions of BRMS1, Keratin19, Orai1, Stim1, TGF-β and VEGF mRNAs in MDA-MB-231 cells. These results suggested that LOXL2 and E-cadherin might play vital roles in the escin Ia-mediated inhibition of MDA-MB-231 cells invasion.

### Escin Ia suppressed epithelial-mesenchymal transition (EMT) in MDA-MB-231 cells and TNF-α/TGF-β-stimulated MCF-7 cells

**Figure 4:**
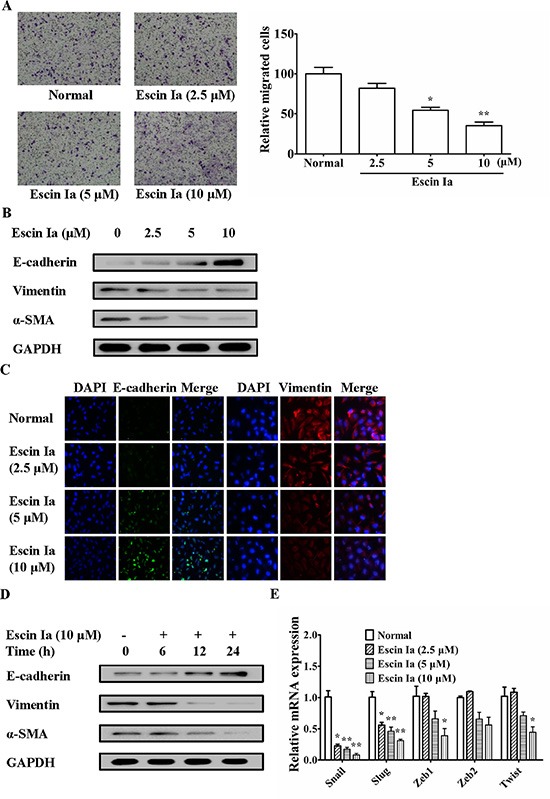
Effect of escin Ia on epithelial-mesenchymal transition in MDA-MB-231 cells MDA-MB-231 cells were treated with escin Ia (2.5, 5, 10 μM) for the indicated intervals. (**A**) Cell migration was detected by using cell migration assay. The number of cells migrated through the bottom chambers were counted in three different regions. (**B**) The protein expressions of E-cadherin, vimentin and α-SMA were detected by using western blot analysis. (**C**) The protein expressions of E-cadherin and vimentin were detected by using immunofluorescence assay. (**D**) Time course of escin Ia on protein expressions of E-cadherin, vimentin and α-SMA were detected by using western blot analysis. (**E**) The mRNA expressions of Snail, Slug, Zeb1, Zeb2 and Twist were detected by using Q-PCR assays. Gene expressions were normalized to GAPDH. The data were expressed as the means ± S.E.M. of three independent experiments. *p < 0.05, **p < 0.01 *vs*. normal.

LOXL2 and E-cadherin are thought to have close relevance to EMT process which could promote invasion and migration of TNBC cells. In addition, escin Ia treatment obviously elevated the mRNA expression of E-cadherin, a well-known marker of the EMT. As shown in Figure 4A, escin Ia obviously inhibited the migration of MDA-MB-231 cells. Furthermore, escin Ia elevated the protein expression of E-cadherin and reduced the protein expressions of vimentin and α-SMA, other two markers of EMT, in MDA-MB-231 cells (Figure 4B and 4C). Of note, escin Ia (10 μM) increased the protein expression of E-cadherin and reduced the protein expressions of vimentin and α-SMA in a time-dependent manner (Figure 4D). Escin Ia (5, 10 μM) down-regulated the mRNA expressions of transcription factors Snail, Slug, Zeb1, Zeb2 and Twist in MDA-MB-231 cells, even though the pattern and degree of these alterations was somewhat different (Figure 4E). Overall, these results strongly suggested that escin Ia could diminish EMT process in MDA-MB-231 cells.

**Figure 5:**
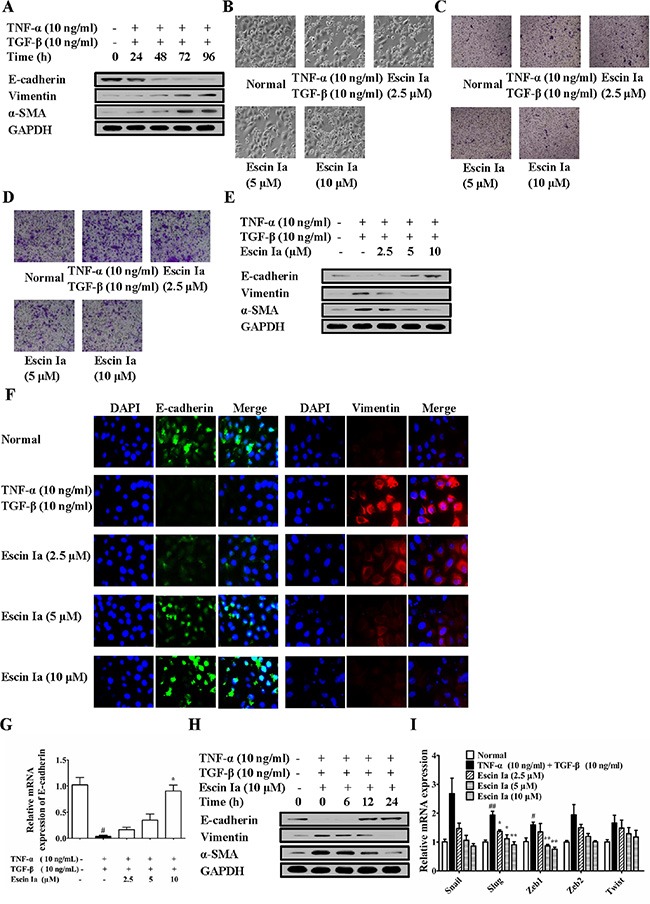
Effect of escin Ia on epithelial-mesenchymal transition in TNF-α/TGF-β-stimulated MCF-7 cells (**A**) MCF-7 cells were treated with TNF-α+TGF-β (10 ng/mL of each) for 0 h, 24 h, 48 h, 72 h, 96 h, and the total protein lysates were immunoblotted for E-cadherin, vimentin and α-SMA expressions. B-I, MCF-7 cells were incubated with TNF-α+TGF-β (10 ng/mL of each) plus escin Ia (2.5, 5, 10 μM) or escin Ia (10 μM) for the indicated internals. Cell morphology was recorded under an inverted microscope (magnification 200 ×) (**B**). Cell invasion was detected by using cell invasion assays (**C**). Cell migration was detected by using cell migration assay (**D**). The protein expressions of E-cadherin, vimentin and α-SMA were detected by using western blot analysis (**E**). The protein expressions of E-cadherin and vimentin were detected by using immunofluorescence assay (**F**). The mRNA expressions of E-cadherin, Snail, Slug, Zeb1, Zeb2, Twist were detected by using Q-PCR assay (**G**, **I**). Gene expressions were normalized to GAPDH. Time course of escin Ia on protein expressions of E-cadherin, vimentin and α-SMA were detected by using western blot analysis (**H**). The data were expressed as the means ± S.E.M. of three independent experiments. ^#^*p* < 0.05, ^##^*p* < 0.01 *vs*. normal; *p < 0.05, **p < 0.01 *vs*. TNF-α (10 ng/mL) + TGF-β (10 ng/mL).

Subsequently, we further investigated the anti-EMT effect of escin Ia in TNF-α/TGF-β-stimulated MCF-7 cells. When stimulated with TNF-α/TGF-β, MCF-7 cells gradually vanished epithelial characteristics and obtained mesenchymal characteristics, and got similar highly aggressive ability as MDA-MB-231 cells [16]. As shown in Figure 5A, after stimulated by TNF-α/TGF-β, the protein levels of E-cadherin and vimentin, α-SMA in MCF-7 cells reached valley and peak, respectively. Escin Ia (2.5, 5, 10 μM) prevented the morphologic transition of MCF-7 cells from epithelial-like to mesenchymal-like appearance (Figure 5B). As shown in Figure 5C and 5D, TNF-α/TGF-β stimulation induced obvious invasion and migration of MCF-7 cells, and escin Ia prohibited these processes. Escin Ia reversed the reduction of the protein and mRNA expression of E-cadherin, and reversed the increase of the protein expressions of vimentin and α-SMA caused by TNF-α/TGF-β stimulation (Figure 5E–5G). The effect of escin Ia (10 μM) gradually increased with exposure time extending (Figure 5H). Escin Ia (5, 10 μM) diminished TNF-α/TGF-β-induced increase of the mRNA expressions of Snail, Slug, Zeb1, Zeb2 and Twist (Figure 5I). Taken together, these results indicated that escin Ia could reverse the EMT process in TNF-α/TGF-β-stimulated MCF-7 cells.

In a word, these findings suggested that EMT repression was an important mechanism for escin Ia-induced inhibition of the invasion of TNBC cells.

### Escin Ia suppressed EMT process via down-regulating LOXL2

**Figure 6:**
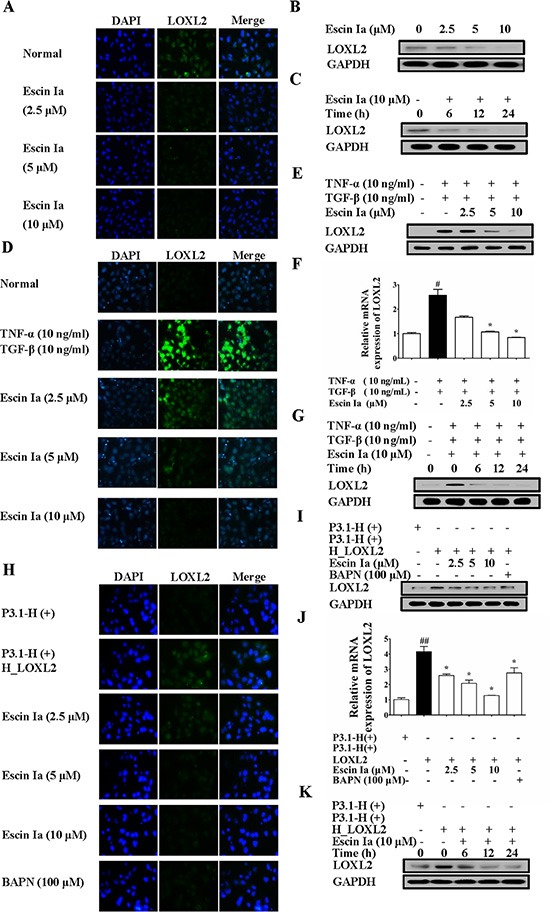
Effect of escin Ia on LOXL2 expression in MDA-MB-231 cells and TNF-α/TGF-β-stimulated or LOXL2-transfected MCF-7 cells (**A**–**C**) Effect of escin Ia on LOXL2 expression in MDA-MB-231 cells. MDA-MB-231 cells were treated with escin Ia (2.5, 5, 10 μM) or escin Ia (10 μM) for the indicated intervals. The protein expression of LOXL2 was detected by using immunofluorescence and western blot analysis (A, B). Time course of escin Ia on protein expression of LOXL2 was detected using western blot analysis (C); D-G, MCF-7 cells were incubated with TNF-α+TGF-β (10 ng/mL of each) plus escin Ia (2.5, 5, 10 μM) or escin Ia (10 μM) for the indicated internals. The protein and mRNA expression of LOXL2 was detected by using immunofluorescence, western blot and Q-PCR assays (**D**–**F**). Time course of escin Ia on protein expression of LOXL2 was detected using western blot analysis (**G**); H-K, MCF-7 cells were transiently transfected with a plasmid of LOXL2 (P3.1-H (+) H_LOXL2) plus escin Ia (2.5, 5, 10 μM), BAPN (100 μM) or escin Ia (10 μM) for the indicated internals. The protein expression of LOXL2 was detected by using immunofluorescence assays, western blot analysis and Q-PCR assays (**H**–**J**). Time course of escin Ia on protein expression of LOXL2 was detected by using western blot analysis (**K**). The data were expressed as the means ± S.E.M. of three independent experiments. ^#^*p* < 0.05 *vs*. TNF-α (10 ng/mL) + TGF-β (10 ng/mL), ^##^*p* < 0.01 *vs*. p3.1-H (+); *p < 0.05 *vs*. TNF-α (10 ng/mL)+TGF-β (10 ng/mL), *p < 0.05 *vs*. p3.1-H(+) H_LOXL2.

LOXL2, a member of the lysyl oxidase family, has been identified as a vital promoting factor of EMT [17], and its mRNA expression was obviously reduced by escin Ia treatment in MDA-MB-231 cells. Moreover, escin Ia could suppress EMT process in MDA-MB-231 cells and TNF-α/TGF-β-stimulated MCF-7 cells. Therefore, we subsequently investigated whether inhibition of EMT by escin Ia was mediated by LOXL2. Firstly, we observed whether escin Ia inhibited LOXL2 expression in breast cancer cells. As shown in Figure 6A–6C, obvious LOXL2 protein expression was observed in MDA-MB-231 cells, and escin Ia (2.5, 5, 10 μM) decreased LOXL2 protein expression. Moreover, TNF-α/TGF-β stimulation for 72 h resulted in a dramatically elevated protein and mRNA expression of LOXL2 in MCF-7 cells, which were abrogated by escin Ia treatment (Figure 6D–6G). In contrast, escin Ia showed little effect on LOXL2 expression and EMT process in MCF-7 cells (Figure S3). On the other hand, MCF-7 cells showed obvious LOXL2 expression, and exhibited a highly aggressive profile similar to MDA-MB-231 cells when transfected with LOXL2 [18]. Escin Ia and BAPN (a non-selective LOXL2 inhibitor) decreased LOXL2 expression of LOXL2-transfected MCF-7 cells (Figure 6H–6K). These data in association with the fact that LOXL2 expression was low in MCF-7 cells suggested that the inhibition of EMT of escin Ia in breast cancer cells correlated with the reduction of LOXL2 expression.

**Figure 7:**
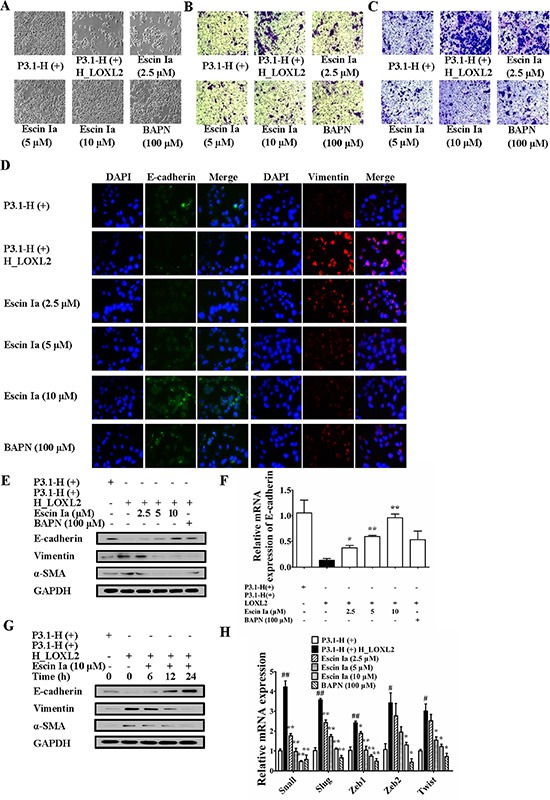
Effect of escin Ia on epithelial-mesenchymal transition in LOXL2-transfected MCF-7 cells MCF-7 cells were transfected with a plasmid of LOXL2 (P3.1-H (+) H_LOXL2) plus escin Ia (2.5, 5, 10 μM) or escin Ia (10 μM) for the indicated internals. (**A**) Cell morphology was recorded under an inverted microscope (magnification 200 ×). (**B**) Cell invasion was detected by using cell invasion assay. (**C**) Cell migration was detected by using cell migration assay. (**D**) The protein expressions of E-cadherin and vimentin were detected by using immunofluorescence assay. (**E**) The protein expressions of E-cadherin, vimentin and α-SMA were detected using western blot analysis. (**F**, **H**) The mRNA expressions of E-cadherin, Snail, Slug, Zeb1, Zeb2 and Twist were detected using Q-PCR assays. Gene expressions were normalized to GAPDH. (**G**) Time course of escin Ia on protein expressions of E-cadherin, vimentin and α-SMA were detected by using western blot analysis. The data were expressed as the means ± S.E.M. of three independent experiments. ^#^*p* < 0.05, ^##^*p* < 0.01 *vs*. p3.1-H(+); *p < 0.05, **p < 0.01 *vs*. p3.1-H(+) H_LOXL2.

In order to confirm that escin Ia inhibited EMT process through down-regulating LOXL2, we observed that effect of escin Ia on EMT process of MCF-7 cells transfected with LOXL2. Results showed that MCF-7 cells gradually vanished epithelial characteristics and obtained mesenchymal characteristics, and exhibited a highly aggressive profile as MDA-MB-231 cells when transfected with LOXL2. As shown in Figure 7A–7C, escin Ia (2.5, 5, 10 μM) and BAPN (100 μM) treatments prevented the morphologic transition of LOXL2-transfected MCF-7 cells from epithelial-like to mesenchymal-like appearance, and inhibited invasion and migration of LOXL2-transfected MCF-7 cells. MCF-7 cells transfected with LOXL2 showed decreased protein and mRNA expression of E-cadherin and elevated protein expressions of vimentin, α-SMA, and escin Ia (2.5, 5, 10 μM) and BAPN (100 μM) antagonized LOXL2-transfection-mediated down-regulation of E-cadherin at protein and mRNA grades, and abrogated LOXL2-transfection-induced up-regulation of vimentin and α-SMA at protein levels (Figure 7D–7G). In addition, escin Ia (5, 10 μM) and BAPN (100 μM) markedly decreased LOXL2-transfection-induced increase of the mRNA expressions of Snail, Slug, Zeb1, Zeb2 and Twist (Figure 7H). These findings indicated that escin Ia inhibited EMT process mainly through down-regulating LOXL2.

**Figure 8:**
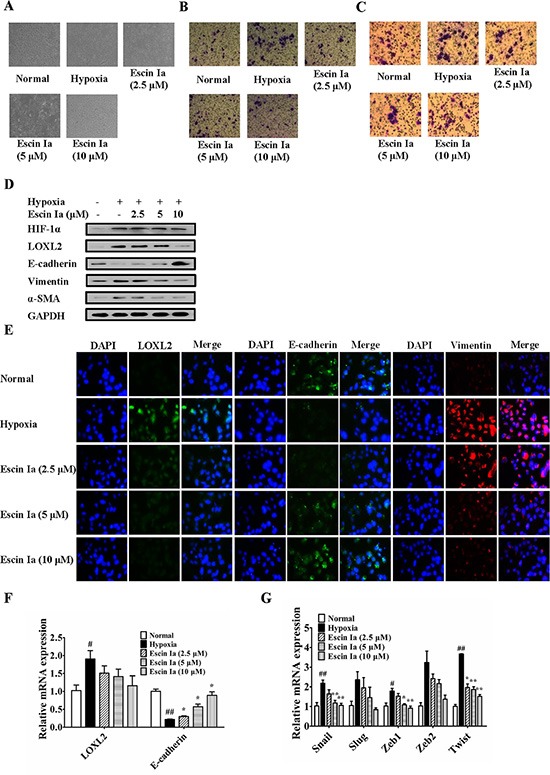
Effect of escin Ia on epithelial-mesenchymal transition in hypoxia-stimulated MCF-7 cells MCF-7 cells were incubated with hypoxia plus escin Ia (2.5, 5, 10 μM) for the indicated internals. (**A**) Cell morphology was recorded under an inverted microscope (magnification 200 ×). (**B**) Cell invasion was detected by using cell invasion assay. (**C**) Cell migration was detected by using cell migration assay. (**D**) The protein expressions of HIF-1α, LOXL2, E-cadherin, vimentin and α-SMA were detected by using western blot analysis. (**E**) The protein expressions of LOXL2, E-cadherin and vimentin were detected by using immunofluorescence assay. (**F**, **G**) The mRNA expressions of LOXL2, E-cadherin, Snail, Slug, Zeb1, Zeb2 and Twist were detected by using Q-PCR assay. Gene expressions were normalized to GAPDH. The data were expressed as the means ± S.E.M. of three independent experiments. ^#^*p* < 0.05, ^##^*p* < 0.01 *vs*. normal; *p < 0.05, **p < 0.01 *vs*. hypoxia.

Furthermore, the anti-EMT effect of escin Ia in hypoxia-stimulated MCF-7 cells was observed. Data showed that LOXL2 level in MCF-7 cells was elevated after the cells were exposed to hypoxia. Epithelial and mesenchymal characteristics of MCF-7 cells gradually vanished and obtained, respectively. In addition, hypoxia resulted in high aggressivity of MCF-7 cells similar to MDA-MB-231 cells [19]. As shown in Figure 8A–8C, escin Ia (2.5, 5, 10 μM) prevented the morphologic transition from epithelial-like to mesenchymal-like appearance, and prohibited the invasion and migration of hypoxia-induced MCF-7 cells. Obvious elevation of the protein and mRNA expressions of HIF-1α, LOXL2, vimentin, α-SMA and decrease of protein and mRNA expression of E-cadherin were observed in hypoxia-stimulated MCF-7 cells, and escin Ia (5, 10 μM) abrogated hypoxia-induced changes of LOXL2, vimentin, α-SMA and E-cadherin expressions (Figure 8D–8F), but it showed little effect on the expression of HIF-1α (Figure 8D). In addition, escin Ia (5, 10 μM) markedly decreased hypoxia-induced increase of the expressions of Snail, Slug, Zeb1, Zeb2 and Twist mRNAs (Figure 8G). These findings indicated that escin Ia inhibited EMT process through down-regulating LOXL2 expression.

### Escin Ia inhibited lung metastasis and EMT process in MDA-MB-231 xenograft model

**Figure 9:**
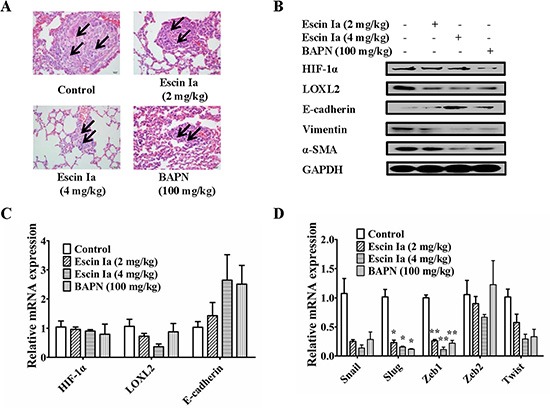
Effect of escin Ia on lung metastasis of MDA-MB-231 xenograft model *in vivo* Mice were injected with MDA-MB-231 cells at the left and right mammary fat pads, and then treated with escin Ia (2, 4 mg/kg) and BAPN (100 mg/kg) for five weeks. (**A**) H & E staining was used for detecting metastatic tumor cells in mouse lungs. Arrows indicated infiltration of tumor cells. (**B**) Tumor samples from the breasts of mice were collected at the end of five weeks and subjected to western blot analysis for HIF-1α, LOXL2, E-cadherin, vimentin and α-SMA expressions. (**C**, **D**) Tumor samples from breast of mice were collected at the end of five weeks and subjected to Q-PCR assays for HIF-1α, LOXL2, E-cadherin, Snail, Slug, Zeb1, Zeb2 and Twist mRNA expressions. Gene expressions were normalized to GAPDH. The data were expressed as the means ± S.E.M. of three independent experiments. *p < 0.05, **p < 0.01 *vs*. control.

To confirm the inhibitory effect on EMT and consequent anti-metastasis activity of escin Ia, a xenograft model was established by subcutaneous injection of MDA-MB-231 cells into the left and right mammary fat pads of nude mice. The metastasis from the injection sites to the lungs was studied by histological examination at seven weeks after MDA-MB-231 cells injection. The infiltration of tumor cells was observed in the lungs of mice in model group. However, no or less infiltration was detected in the lungs of escin Ia- and BAPN-treated mice. Of note, the potency of escin Ia (4 mg/kg) was superior to that of BAPN (100 mg/kg) (Figure 9A).

Tumor samples from the breasts of mice in model group, escin Ia (2 mg/kg, 4 mg/kg)-treated groups and BAPN (100 mg/kg)-treated groups were used to determine the effect of escin Ia on the expressions of HIF-1α, LOXL2 and EMT markers. It was shown that the expression of E-cadherin was lower, and the expressions of HIF-1α, LOXL2, vimentin, α-SMA, Snail, Slug, Zeb1, Zeb2 and Twist were higher in the tumors in the model group. Escin Ia (4 mg/kg) and BAPN (100 mg/kg) treatments caused down-regulations of LOXL2, vimentin, α-SMA, Snail, Slug, Zeb1, Zeb2 and Twist and up-regulation of E-cadherin at protein and mRNA grades in MDA-MB-231 xenografts mice (Figure 9B–9D). However, escin Ia (2 mg/kg, 4 mg/kg) showed little effect on protein and mRNA expression of HIF-1α in breast tumors of mice (Figure 9B and 9C). Taken together, the findings indicted that escin Ia suppressed the TNBC metastasis by inhibition of EMT process and consequent invasive growth through down-regulating LOXL2.

## DISCUSSION

The two primary hallmarks of TNBC are excessive proliferation and high metastasis of cancer cells. Increasing evidence has shown that the metastasis of TNBC cells mainly involves invasion, migration and angiogenesis, and the invasion is considered as the key step in metastasis process. SFAC markedly inhibited the invasion of MDA-MB-231 cells, and its anti-invasion potency was superior to that of inhibition of proliferation, apoptosis, migration and angiogenesis. It was suggested that invasion inhibition was the major mechanism for SFAC suppressing TNBC metastasis. In the present study, escin Ia, which showed stronger inhibition of the invasion of MDA-MB-231 cells than other five saponin ingredients isolated from SFAC, was proven to be the major anti-invasion active constituent of SFAC.

Invasion can be regulated by a variety of factors such as BRMS1, E-cadherin, Keratin19, LOXL2, MMP9, Orai1, Stim1, TGF-β and VEGF. These factors with lower or higher expressions are associated with poor prognosis of TNBC patients. Therefore, the abrogation of the expressions of the aberrant factors may inhibit invasion and TNBC metastasis, and can be considered as a good strategy for TNBC therapy [20–26]. Our study showed that escin Ia obviously inhibited invasion of MDA-MB-231 cells *in vitro*, and prevented lung metastasis of MDA-MB-231 xenograft model *in vivo*. In addition, escin Ia markedly decreased mRNA expression of LOXL2 and increased mRNA expression of E-cadherin but not the other invasion-related factors, which indicated that LOXL2 and E-cadherin are important in escin Ia-mediated inhibition of MDA-MB-231 cell invasion.

EMT is a developmental and continuous process, and has been identified as the beginning of metastasis of cancers including TNBC [27]. Indeed, overexpression of EMT markers in breast cancer biopsies correlates with tumor aggressiveness, adverse clinicopathological characteristics, increased recurrence, and shorter survival. Therefore, it may be of great therapeutic interest to develop effective therapeutic strategies to suppress EMT in TNBC cells to prevent its invasion. EMT process can be promoted by a lot of important transcription factors, such as Snail, Slug, Zeb1, Zeb2 and Twist [28]. These factors with higher expression are associated with poor prognosis of TNBC patients. In addition, the factors are highly expressed in MDA-MB-231 cells. Thus, reduction of the factors is a good method for EMT inhibition [29–35]. Our present study showed that EMT process of MDA-MB-231 cells and TNF-α/TGF-β-stimulated MCF-7 cells were significantly inhibited by escin Ia treatment. In addition, escin Ia suppressed EMT process in MDA-MB-231 xenograft model. These findings indicated that EMT inhibition was an important mechanism for the anti-invasion effect of escin Ia on TNBC.

LOXL2 encodes an extracellular copper-dependent amine oxidase, and it is over-expressed in various types of primary cancer tissues and cells. Importantly, increasing evidence indicates that high LOXL2 expression correlates with tumor grade and poor survival. Therefore, reduction of LOXL2 expression may be an effective approach for TNBC therapy [36–38]. Our data showed that escin Ia markedly decreased LOXL2 expression of MDA-MB-231 cells, and abrogated the increase of LOXL2 expression induced by TNF-α/TGF-β stimulation, LOXL2 transfection or hypoxia in MCF-7 cells. Furthermore, LOXL2 expression was also down-regulated by escin Ia treatment in MDA-MB-231 xenograft model. However, escin Ia showed slight effect on LOXL2 expression and EMT process in MCF-7 cells. It was a remarkable fact that LOXL2 but not E-cadherin, vimentin, α-SMA expressions was down-regulated by escin Ia treatment for 6 h in MDA-MB-231 cells and MCF-7 cells treated with TNF-α/TGF-β. These findings suggested that escin Ia might suppress EMT process through LOXL2. Based on these findings, we further investigated the relationship between the reduction of LOXL2 expression and inhibition of EMT process by escin Ia. The results showed that EMT process of LOXL2-transfected MCF-7 cells was significantly inhibited by escin Ia treatment for 6 h, which was accompanied by a decrease of the expression of LOXL2 but not E-cadherin, vimentin and α-SMA. Moreover, escin Ia markedly reduced LOXL2 expression and suppressed EMT process in hypoxia-stimulated MCF-7 cells. These findings strongly suggested that escin Ia suppressed EMT process through LOXL2, and resulted in the inhibition of TNBC invasion.

HIF-1α, a master transcriptional regulator of cellular and developmental response to hypoxia, was proven to facilitate invasion and metastatic niche formation [39]. In MDA-MB-231 cells, knockdown of HIF-1α blocked hypoxia-induced expression of LOXL2, which indicated that HIF-1α located at the upstream of LOXL2 [40]. Our present data showed that escin Ia showed little effect on HIF-1α expression in MDA-MB-231 cells (data not shown) and hypoxia-stimulated MCF-7 cells. Consistently, escin Ia showed slight effect on HIF-1α expression in MDA-MB-231 xenograft mice.

In conclusion, escin Ia is the major active constituent of SFAC for its inhibitory effect on the invasion of TNBC, and it functions by inhibition of EMT process *via* down-regulating LOXL2 expression.

## MATERIALS AND METHODS

### Reagents

SFAC (containing saponin constituents higher than 74.4%), escin Ia (C_55_H_86_O_24_, MW: 1131.26, purity ≥ 98%), escin Ib (C_55_H_86_O_24_, MW: 1131.26, purity ≥ 98%), escin IIa (C_54_H_84_O_23_, MW: 1101.23, purity ≥ 98%), escin IIb (C_54_H_84_O_23_, MW: 1101.23, purity ≥ 98%), escin IIIa (C_55_H_86_O_23_, MW: 1115.26, purity ≥ 98%) and escin IIIb (C_55_H_86_O_23_, MW: 1115.26, purity ≥ 98%) were obtained from Yinxing Pharmaceutical Technology Co., Ltd. (Nanjing, China) (Figure 1); β-aminopropionitrile (BAPN) was obtained from Sa'en Co., Ltd. (Shanghai, China); Transforming growth factor-β (TGF-β) and tumor necrosis factor-α (TNF-α) were obtained from R & D Systems (Minneapolis, USA); TRIzol and Lipofectamine 2000 reagents were obtained from Invitrogen (Carlsbad, USA); E-cadherin and LOXL2 antibodies were purchased from Genetex (Irvine, USA); HIF-1α and vimentin antibodies were purchased from BioWorld (Georgia, USA); α-SMA antibody was purchased from Abcam (Cambridge, UK); GAPDH monoclonal antibody was purchased from KangChen Bio-tech (Shanghai, China); HRP-conjugated secondary antibodies were purchased from Abbkine (Redlands, USA); Fetal bovine serum (FBS) was obtained from PAA (Linz, Germany). Other analytical reagent grade chemicals were obtained from Sinopharm Chemical Reagent Co. Ltd (Nanjing, China).

### Cell culture

MDA-MB-231 cells and MCF-7 cells were obtained from American Type Culture Collection (ATCC, Manassas, VA, USA), and cultured in a humidified incubator at 37°C under 5% CO_2_ atmospheric condition in corresponding medium Supplementaryed with 10% FBS, 100 U/mL streptomycin and 100 U/mL penicillin.

### Cell transfection

The plasmid expressing a constitutively active form of LOXL2 was purchased from Jiman Biochemical Technology Co., Ltd (Shanghai, China). MCF-7 cells were transfected with LOXL2 by using Lipofectamine 2000 (Invitrogen, Carlsbad, USA) according to manufacturer's instructions.

### Cell migration assay

Cell migration assay was carried out by using transwell plates (Millipore, Billerica, USA) as described previously with a little modification. MDA-MB-231 cells and MCF-7 cells were transfected with LOXL2, stimulated with TNF-α/TGF-β or hypoxia (a modified incubator chamber flushed with a gas mixture containing 1% O_2_, 94% N_2_ and 5% CO_2_ in a humidified atmosphere). Then, they were treated with escin Ia (2.5, 5, 10 μM) or BAPN (100 μM) for 6 h, and detached and suspended in culture medium. Then, cells (1 × 10^4^ cells/well) was added into the upper chamber of the transwell plates, while the lower chamber was full of 600 μL culture medium with 10% FBS as a chemoattractant. After being incubated for 6 h at 37°C, the non-migrated cells on the upper surface of the membrane were removed by soaked cotton swab. In addition, the cells migrated to the bottom face of the membranes were counted after being stained with crystal violet solutions. Then, fields per filter were captured randomly at a magnification of 200 × with Olympus IX51 inverted microscope.

### Tube formation assay

Matrigel (150 μL/well) was added to 48-well plates and allowed to polymerize for 1 h. Human umbilical vein endothelial cells (HUVECs, MaiGaoQiao Hospital, Nanjing, China) were suspended and added into each well (8 × 10^4^ cells/well) together with indicated concentrations of SFAC. The capillary tube formations were visualized after 24 h. The images were captured by an Olympus IX51 inverted microscope using a 100 × objective lens. The tube formation was defined by measuring the branch length of the formed tubes.

### Cell invasion assay

Cell invasion assay was performed by using transwell chamber with 10 mm diameter and 8 μm pore size polycarbonate membrane (Corning Costar, Cambridge, UK) coated with matrigel as previously described. MDA-MB-231 cells and MCF-7 cells, transfected with LOXL2 or stimulated with TNF-α/TGF-β or hypoxia, were treated with escin Ia (2.5, 5, 10 μM) or escin Ib-IIIb (5 μM) or BAPN (100 μM) for 24 h, and detached and suspended in culture medium. An aliquot (200 μL) of cells (1 × 10^5^ cells/mL) was added into the upper chamber of the transwell, while the lower chamber was full of 600 μL medium with 10% FBS as a chemoattractant. After being incubated for 24 h at 37°C, the non-invaded cells on the upper surface of the membrane were removed by soaked cotton swab, and the cells invaded to bottom face of the membranes were counted after being stained with crystal violet solutions. Then, fields per filter were captured randomly at a magnification of 200 × with Olympus IX51 inverted microscope.

### Quantitative real-time polymerase chain reaction (Q-PCR)

MDA-MB-231 cells, MCF-7 cells transfected with LOXL2 or stimulated with TNF-α/TGF-β or hypoxia were treated with escin Ia or BAPN, and the total RNA were isolated by using TRIzol reagent according to the supplier's instructions. The cDNA was transcribed from RNA by using HiScript RT Super Mix (Vazyme, Nanjing, China), then analyzed for the expressions of Snail, Slug, Zeb1, Zeb2, Twist, LOXL2, hypoxia-inducible factor 1-alpha (HIF-1α), E-cadherin, breast cancer metastasis suppressor 1 (BRMS1), metal matrix proteinase 9 (MMP9), calcium release-activated calcium channel protein 1 (Orai1), stromal interaction molecule 1 (Stim1), Keratin19, TGF-β and VEGF by Ace Q-PCR SYBR Green Master Mix (Vazyme, Nanjing, China) with the help of MyiQ2 Detection System (Bio-Rad Laboratories, Hercules, USA). The primer sequences used were listed in Table 1:

**Table 1:** Primer pairs used in quantitative real-time PCR

Gene		sequence (5′-3′)	Length (bp)
GAPDH	forward	AGAAGGCTGGGGCTCATTTG	258
	reverse	AGGGGCCATCCACAGTCTTC	
VEGF	forward	CTACCTCCACCATGCCAAGT	187
	reverse	CACACAGGATGGCTTGAAGA	
MMP9	forward	CTACCACCTCGAACTTTGAC	161
	reverse	CTCAGTGAAGCGGTACATAG	
TGF-β	forward	GGCCCTGCCCCTACATTT	75
	reverse	CCGGGTTATGCTGGTTGTACA	
E-cadherin	forward	GACCGAGAGAGTTTCCCTACG	158
	reverse	TCAGGCACCTGACCCTTGTA	
Keratin19	forward	GATGAGCAGGTCCGAGGTTAC	106
	reverse	TGCCAGTGTGTCTTCCAAGG	
Orai1	forward	ACGTGCACAATCTCAACTCG	121
	reverse	AGCACCACCTCAGCTAGGAAG	
Stim1	forward	CTCCTCTCTTGACTCGCCAT	154
	reverse	CGGTGTAACCCCTCCAAGTC	
LOXL2	forward	GTGGATCTGGCACGACTGTCA	75
	reverse	TTGAGGTTCAGCAGGTCATAGTGG	
BRMS1	forward	ATGCCTGTCCAGCCTCCAAG	168
	reverse	GCGTCGCTCATAGTCCTCATCA	
HIF-1α	forward	CCCATTAGCAGGTGAAGGAA	141
	reverse	CCAGAATCAAACCAAACCAA	
Snail	forward	CCCCAATCGGAAGCCTAACT	71
	reverse	CGTAGGGCTGCTGGAAGGTA	
Slug	forward	CCATTCCACGCCCAGCTA	71
	reverse	CTCACTCGCCCCAAAGATGA	
Twist	forward	GGAGTCCGCAGTCTTACGAG	201
	reverse	TCTGGAGGACCTGGTAGAGG	
Zeb1	forward	ATGCGGAAGACAGAAAATGG	297
	reverse	GTCACGTTCTTCCGCTTCTC	
Zeb2	forward	AACAACGAGATTCTACAAGCCTC	176
	reverse	TCGCGTTCCTCCAGTTTTCTT	

### Western blotting

MDA-MB-231 cells and MCF-7 cells transfected with LOXL2 or stimulated with TNF-α/TGF-β or hypoxia were treated with escin Ia or BAPN, and the total cell lysates were prepared by using NP40 buffer (Beyotime, Nanjing, China). The equal concentration of protein lysate of all the samples was separated on 8% SDS-PAGE gel and further transferred to PVDF membranes. The membranes were blocked with 5% non-fat milk for 2 h, and incubated with specific primary antibodies overnight at 4°C. After being washed with TBS-0.1% Tween, membranes were incubated with HRP-conjugated anti-mouse or anti-rabbit IgG secondary antibodies, and then visualized with ECL reagent (Guge Biochemical Technology Co., Ltd., Wuhan, China).

### Immunofluorescence assay

MDA-MB-231 cells and MCF-7 cells (2 × 10^3^ cells/mL) transfected with LOXL2, stimulated with TNF-α/TGF-β or hypoxia, were treated with escin Ia or BAPN, and then detached, re-suspended and plated into 96-well chamber slides (Millipore, Billerica, USA). Cells were fixed and stained after incubation according to the supplier's instructions.

### Animals and treatment

Female athymic nude mice, 4–6 week-old, were obtained from Cavens Laboratory Animals Co., Ltd. (Changzhou, China). All animal experiments were performed in strict accordance with the Guide for the Care and Use of Laboratory Animals.

MDA-MB-231 cells re-suspended in PBS (1 × 10^7^ cells/mL) were injected subcutaneously into the left and right mammary fat pads of mice. Two weeks after initial implantation, mice were divided into four groups: 1) control group; 2) escin Ia (2 mg/kg) group; 3) escin Ia (4 mg/kg) group; 4) BAPN (100 mg/kg) group. Escin Ia and BAPN were intraperitoneally injected three times per week for the duration of the experiment.

All animals were sacrificed after five weeks of treatments, and the tumors from the breasts of mice were collected and utilized for the detection of RNA and protein by using Q-PCR and western blotting assays, respectively.

### Statistical analysis

The data were presented as the means ± S.E.M. Statistical analysis was performed by using one-way analysis of variance (ANOVA) followed by Tukey's test. *P* values less than 0.05 (*P* < 0.05) were accepted as a significant difference.

## SUPPLEMENTARY MATERIALS FIGURES


