# Supplementary material for: Escin Ia suppresses the metastasis of triple-negative breast cancer by inhibiting epithelial-mesenchymal transition via down-regulating LOXL2 expression
Source: Oncotarget. 2016 Mar 17;7(17):23684–99. doi: 10.18632/oncotarget.8152 (PMC5029656; doi:10.18632/oncotarget.8152)
Supplement: Supplementary file 1 [file oncotarget-07-23684-s001.pdf]

Escin Ia suppresses the metastasis of triple-negative breast cancer by inhibiting epithelial-mesenchymal transition via down-regulating LOXL2 expression

Supplementary Materials

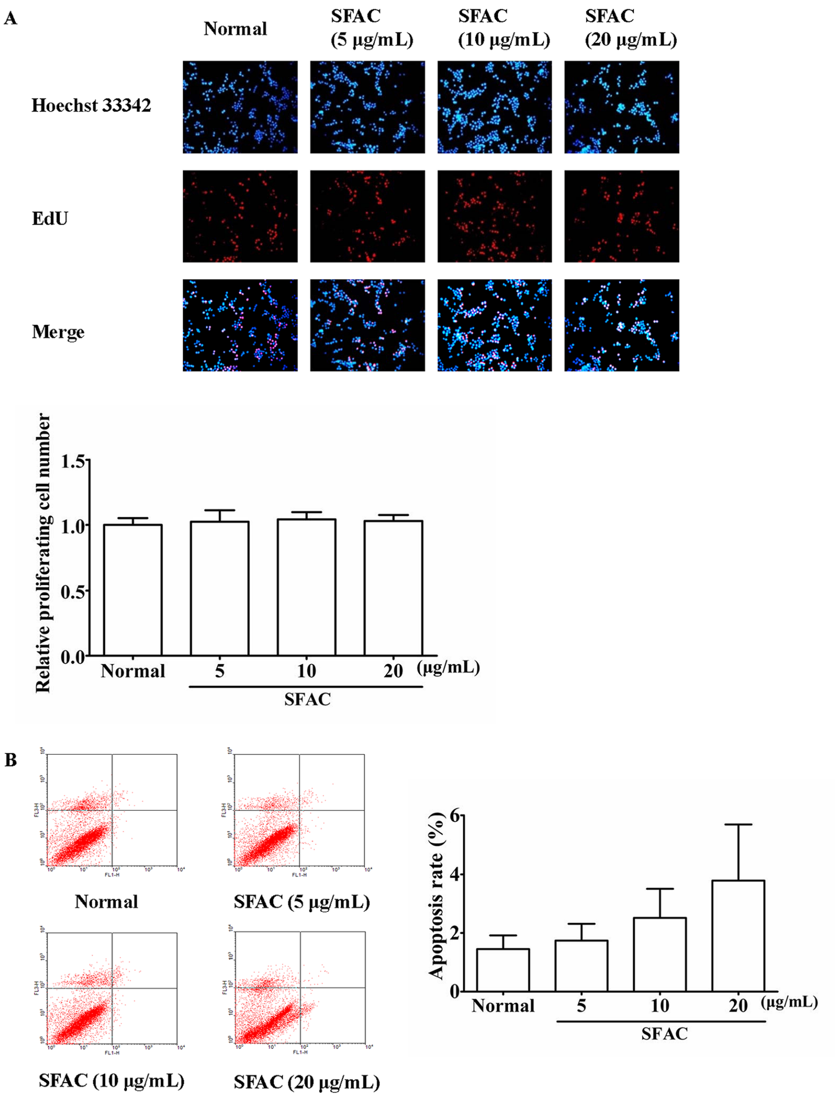

Supplementary Figure S1: Effect of the saponin fraction isolated from *Aesculus chinensis* Bunge fruits (SFAC) on proliferation and apoptosis of MDA-MB-231 cells. (**A**) Cells were stained with 5-ethynyl-2-deoxyuridine (EdU) and Hoechst 33342 after incubation of SFAC at different concentrations for 24 h. Proliferation rates were calculated by normalizing the numbers of EdU-positive cells to Hoechst-stained cells at five random fields (250 × magnification). (**B**) Cell apoptosis was measured by using flow cytometry stained with Annexin V-FITC/PI. Apoptosis rates were quantified. The data were expressed as the means ± S.E.M. of three independent experiments.

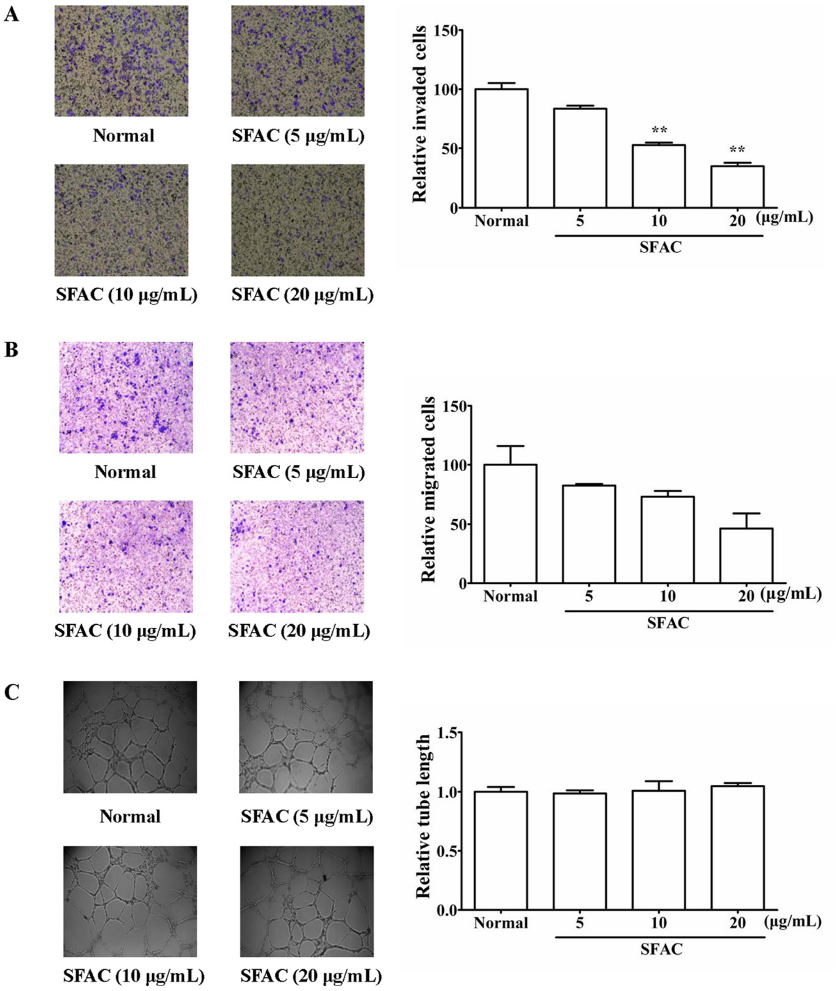

Supplementary Figure S2: Effect of the saponin fraction isolated from *Aesculus chinensis* Bunge fruits (SFAC) on invasion, migration and angiogenesis. (**A**, **B**) Effect of SFAC on invasion and migration of MDA-MB-231 cells. MDA-MB-231 cells were treated with SFAC (5, 10, 20 μg/mL) for the indicated internals. Cell invasion was detected by using cell invasion assay. The number of cells invaded through the bottom chambers were counted in three different regions (A). Cell migration was detected by using cell migration assay. The number of cells migrated through the bottom chambers were counted in three different regions (B). Matrigel was added to 48-well plates and allowed to polymerize for 1 h. (**C**) Human umbilical vein endothelial cells (HUVECs) were added into each well together with treatment of SFAC (5, 10, 20 μg/mL) for 24 h. The capillary tube formations were visualized by an inverted Olympus IX51 inverted microscope using a 100 × objective lens. Tube length of each group was quantified. The data were expressed as the means ± S.E.M. of three independent experiments. *$p < 0.05$, **$p < 0.01$ *vs.* normal.

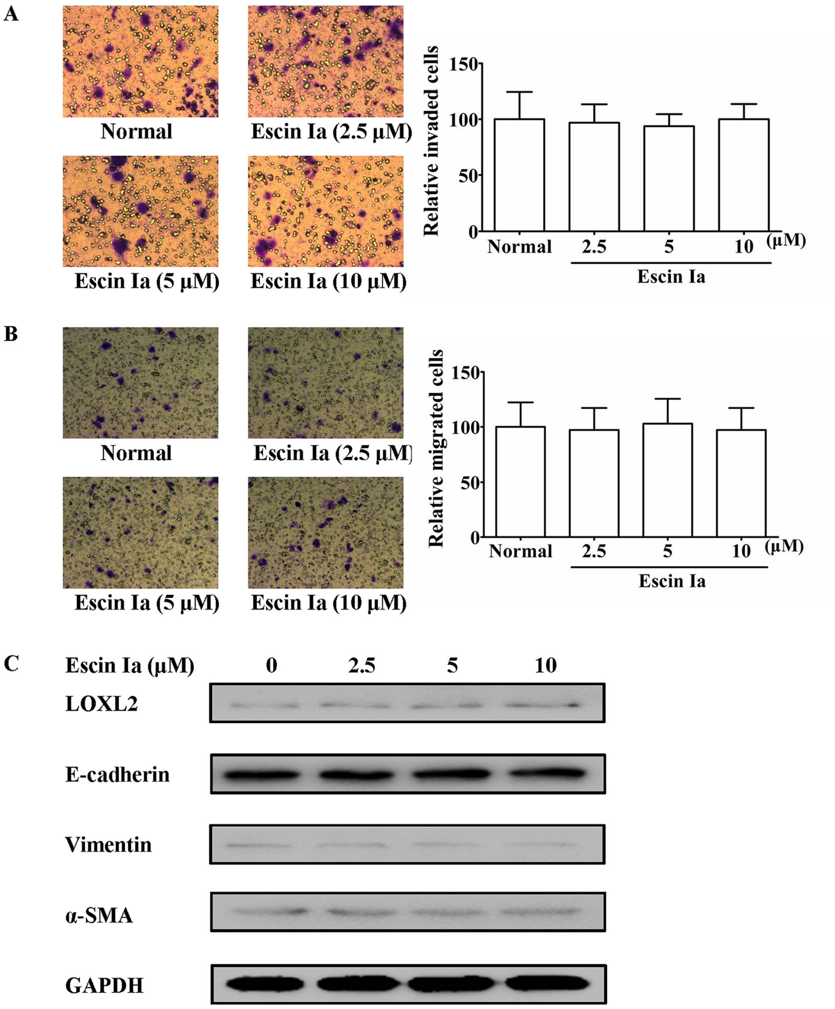

Supplementary Figure S3: Effect of escin Ia on epithelial-mesenchymal transition in MCF-7 cells. MCF-7 cells were treated with escin Ia (2.5, 5, 10 μM) for the indicated intervals. (**A**) Cell invasion was detected using cell invasion assays. The number of cells invaded through the bottom chambers were counted in three different regions. (**B**) Cell migration was detected by using cell migration assays. The number of cells migrated through the bottom chambers were counted in three different regions. (**C**) The protein expressions of LOXL2, E-cadherin, vimentin and α-SMA were detected using western blot analysis. The data were expressed as the means ± S.E.M. of three independent experiments.
